# Alternation preferences affect focus marking in German and English differently

**DOI:** 10.3389/fpsyg.2023.1192004

**Published:** 2023-06-05

**Authors:** Nadja Schauffler

**Affiliations:** Institute of English Linguistics, University of Stuttgart, Stuttgart, Germany

**Keywords:** alternation, focus marking, rhythm rule, stress clash, double focus

## Abstract

This study investigates the interplay between alternation preferences and corrective focus marking in the production of German and English speakers. Both languages prefer an alternation of strong and weak, and both use pitch accenting to indicate focus structure. The objective of the study is to determine whether the preference for rhythmic alternation can account for variations in the prosodic marking of focus. Contrary to previous claims, the results obtained from three production experiments indicate that rhythmic adjustment strategies do occur during focus marking. However, despite the similarities between the two languages, they employ different strategies when alternation and focus marking work in opposite directions. German speakers often employ a melodic alternation of high and low by realizing the first of two adjacent focus accents with a rising pitch accent (L^*^H), while English speakers frequently omit the first focus accent in clash contexts. This finding is further supported by a second experiment that investigates pitch accent clashes in rhythm rule contexts under various focus environments. The findings suggest that the preference for alternation can influence the prosodic marking of focus and contributes to variation in the realization of information-structure categories.

## 1. Introduction

In German and in English, information structure and prosody are highly intertwined in so far as information structure is marked by prosodic events. So it has been claimed that specific information structure categories, such as focus, are marked by both pitch accent placement and with a specific accent type. While this relationship between information structure and prosody is quite straightforward, there is, however, variation. We often find various prosodic realizations of one information-structure category or one prosodic realization for various categories: Sometimes pitch accent types deviate from what would be expected (cf. Baumann, 2006; Féry, 2007; Féry and Kügler, 2008; Schweitzer et al., 2009) and sometimes pitch accents are not realized at all even though there should be one from an information-structural perspective (e.g., Kentner, 2012a; Riester and Piontek, 2015).

The aim of this study is to investigate whether there are prosody-inherent factors that contribute to variation in the prosodic implementation of focus. Specifically, it is explored whether and how a preference for rhythmic alternation affects the prosodic marking of focus in German and English.

With German and English, two well-investigated stress-timed languages are chosen that share a number of similarities. First, German and English prosodically mark focus by means of pitch accent placement and pitch accent type, as already mentioned, but there is variation (see Section 1.1).

Second, German and English prefer an alternation of strong and weak beats. This means they prefer an alternation of stressed and unstressed syllables and try to avoid stress clashes, meaning two directly adjacent stresses, wherever possible. This preference is addressed in Section 1.2. The overall research question of this study is how this preference for rhythmic alternation affects the prosodic marking of corrective focus in German and English, respectively.

### 1.1. Pitch accents and information structure

The first starting point is the prosodic marking of information-structure categories in German and English, such as focus. Both languages use pitch accenting to indicate focus structure, and they have been shown to do so by similar means. While, for example, givenness is typically marked with deaccentuation, a contrastive focus is typically associated with a falling pitch accent with a high tonal target on the accented syllable (H*L, the * indicates the association with the stressed syllable; e.g., Selkirk, 1995; Büring, 1997; Gussenhoven, 2004; Kügler and Gollrad, 2015; Féry, 2017). Phonetically, the pitch accent in a contrastive focus was found to have a high and late F_0_ peak, a great pitch excursion and a long duration compared to pitch accents on constituents in broad focus (see, e.g., Baumann et al., 2007; Kügler and Gollrad, 2015 for German, and Gussenhoven, 2004; Breen et al., 2010 for English). The height of the pitch peak correlates with the level of emphasis—the more explicit the contrast of the focus is, that is the more closed the set of alternatives, the higher the perceived pitch (Gussenhoven, 2004; Kügler and Gollrad, 2015; Grice et al., 2017).

In the experiments at hand, double-focus sentences with corrections are used. Double-focus sentences are sentences involving two foci giving answers to two questions such as “*what* did she give?” and “*to whom* did she give it?”. The answer then involves two foci: *She gave [the piano]*_*Focus*1_ *to [teachers]*_*Focus*2_. In our stimuli design, such an answer is elicited in a context of correction, such as in *She didn't give the drums to students, she gave [the piano]*_*F*_ *to [teachers]*_*F*_.

In terms of the intonation of double-focus sentences in German, Büring (1997) predicts two falling pitch accents on both foci (H*L). In a production experiment, Wang and Féry (2018) found that there are three ways in which the first focus in double-focus sentences^1^ is prosodically realized in German, correlating with sentence length: Short sentences were preferably realized with a rising pitch accent on the first focus and a falling pitch accent on the second (“hat-pattern”). Longer sentences were mostly realized with two falling pitch accents on each of the foci (“two-peak pattern”), or with falling accents on both foci but each in its own intonation phrase with a high boundary tone between them (“two-phrase pattern”). See 1 for an example of a sentence in the short condition.

(1) [Der LEHrer] lobt [MAli]
  the teacher praises Mali

Additionally, they found that double-focus sentences differ from initial-focus sentences in that there is no post-focal compression after the first focus, that is F_0_ is not lowered after the first focus as it is the case in single-focus sentences.

Experimental investigations of double-focus in English were conducted by Eady et al. (1986) and Liu (2010). The former investigated the acoustic realizations of double-focus sentences of the kind given in example (2; the key content words are italicized as taken from the original, the two foci are indicated by the author), and compared them to single-focus sentences. They found that, compared to non-focused items and neutral focus items, narrow focused items had a higher F_0_ peak and were longer in duration, regardless of whether the sentence contained one or two foci. In terms of pitch accent type, the contours of both single and double-focused words were falling. There was no evidence that the two foci in a double-focus context affected each other in any way. Differences between initial focus and the first focus in a double-focus sentence were only found regarding post-focal compression, which was absent in double-focus contexts.

(2) Who shot the puck to whom? [*Don*]_*Focus*1_ shot the *puck* to [*Kent*]_*Focus*2_.

Liu (2010) looked at the realization of double-focus statements and double-focus yes/no questions by four native speakers of General American English. She extended on Eady et al.'s work by additionally manipulating sentence length and the position of the focus constituents. Looking at F_0_ and duration on all syllables, her results, too, showed that F_0_ and duration of the focused words in double-focus sentences were in all positions increased to the same degree as in single-focus sentences. She did not find an effect of sentence length.

To sum up, the two studies show that double focus in English is in a way a composition of two single foci in terms of pitch, duration and contour. This is different from what has been found for German where the presence of the second focus caused various realizations of the preceding first focus (Wang and Féry, 2018).

In the study at hand, the double-focus sentences used most closely correspond to the condition for short sentences by Wang and Féry.

While the relationship between information structure and prosody outlined above is quite persuasive, it is not always completely reliable: sometimes there is variation in the actual choice of pitch accent types. There are numerous studies that demonstrate that the same accent type may be used for different discourse functions and the same information-structural category may be marked by different accent types (as just seen in German double-focus sentences). Grice et al. (2017), for example, investigated the production of broad, narrow and contrastive focus and found that while speakers prefer specific accent types for particular focus types this relationship is rather probabilistic and not exclusive—there is variation both within and across speakers. Variation in pitch accent type has also been found with respect to information status, i.e., the marking of degree of givenness of a referent in the discourse (cf. e.g., Baumann and Grice, 2006). While there is the tendency that novelty is expressed with falling accents and given or accessible information with rising accents or no accents, these categories cannot be unambiguously mapped to these specific accent types (Brown, 1983; Baumann, 2006; Baumann and Grice, 2006; Féry and Kügler, 2008; Schweitzer et al., 2009).

Findings like these have brought Roettger et al., (2019, p. 2) to the conclusion “[...] that there is no one-to-one-mapping between intonational events and speaker intentions; any assumed mapping is probabilistic at best (systematic but not deterministic).” Also Kügler and Genzel (2012) concludes that the function of focus accents is to highlight the focus and that this function is fulfilled by a deviation from neutral register, regardless of which direction the deviation takes.

But variation is not only found in the type of pitch accent used to mark discourse structure but also in the placement of pitch accents in the first place. Various studies found variation with respect to accent placement in experimental as well as corpus data. Speakers sometimes place unexpected pitch accents on, for example, given information (e.g., Yule, 1980; Nooteboom and Kruyt, 1987; Bard and Aylett, 1999) or they omit an expected one, for example, on new information (Terken and Hirschberg, 1994). In their investigation of a German radio news corpus (DIRNDL, Eckart et al., 2012) Riester and Piontek (2015), for instance, found several cases in which an accent was missing where it would have been expected and instead is realized on an earlier syllable. The authors explain this shift with rhythmic preferences: the pitch accent was shifted away from a following pitch accented word in order to avoid two pitch accents directly following each other. Other studies came to similar conclusions. Kentner (2012a), for example, found that in unprepared oral reading, speakers avoided producing an information-structurally required accent when the production would have resulted in a stress clash.

### 1.2. Alternation preferences influence sentence production and processing

Looking for rhythmic factors to explain unexpected pitch accenting is motivated by numerous studies that have shown that speakers—and listeners—prefer an alternation of strong and weak beats and avoid structures that violate this principle (e.g., Quené and Port, 2003; Geiser et al., 2006; Bohn et al., 2011; Breen and Clifton, 2011; Kentner, 2012a; Rothermich et al., 2012; Tilsen, 2012; Kimbell and Cole, 2014). This preference has long been known as the Principle of Rhythmic Alternation describing a tendency not only in language but also in music to create rhythmically well-formed structures (e.g., Sweet, 1876; Liberman and Prince, 1977; Selkirk, 1984; Schlüter, 2005). What is considered well-formed in language may be language-specific—for some languages, it may be the avoidance of stress clashes, for others it may be the preference to stress the first or the last syllable in a phrase (cf. Wagner, 2008). In either case, well-formedness emanates from a certain structure which results in regularly recurring events, thereby creating expectations.

It is reasonable to assume that German and English share similar influences of rhythmic alternation on the language, since they both belong to the group of “stress-timed” languages. In stress-timed languages the distance between stressed syllables is assumed to be isochronous, whereas in languages which belong to the group of “syllable-timed” languages, such as, for example, French, Spanish, or Italian, it is the syllables that are isochronously distributed (Pike, 1945; Abercrombie, 1965, 1967).^2^ While neat isochrony of these respective intervals was not found experimentally in any measurable way (e.g., Pointon, 1980; Roach, 1982; Dauer, 1983) these terms are still regarded as extreme points on a continuum correlating with, for example, the degree of vowel reduction and phonotactic complexity (e.g., Dauer, 1983; Auer and Uhmann, 1988).

In her investigation on the Principle of Rhythmic Alternation in English corpora, Schlüter (2005) found numerous instances where rhythmic alternation preferences affected grammatical variation. To name but one, she found effects of alternation preferences on the use of variants of multiword attributive structures, such as *quite a* vs. *a quite*, where the former was more often used preceding an initially-stressed adjective (e.g., *quite a different view*) thereby avoiding a stress clash, compared to the latter (cf. also Bolinger, 1965). Alternation preferences have also been argued to have influenced the stress shift in words after derivational affixes are added (e.g., *solid* vs. *solidity*), or the different stress patterns of English nouns (mainly trochaic) and English verbs (mainly iambic) as a result of the different rhythmic environments they occur in Kelly (1988) and Kelly and Bock (1988). It has also been shown to affect the choice of dative constructions (Anttila et al., 2010), genitive construction (Shih et al., 2015) or word order (Vogel et al., 2015), and the historical loss of certain word forms (Schlüter, 2005).

For German, Vogel et al. (2015) could show that function words are frequently used in a way which optimizes “the rhythmic flow in speech.” In one study they found, for instance, that pronouns are used in their strong, weak, or reduced form depending on the rhythmic context, which also affects the optional word order in three-verb clusters as shown by another study (Vogel et al., 2015). On a study with German preschoolers and adults, Franz et al. (2021) found that the rhythmic environment affected decisions on word order in a picture-naming task in both groups, such that a sequence of unstressed syllables (stress lapses) was avoided.^3^ The preference for alternation was also found to influence the appearance of optional schwa in German adverbs [e.g., *gern(e)*, “happily”], as found by Kentner (2018). He also found that the rhythmic context affected readers' choice between a monosyllabic and a disyllabic morphological genitive or a disyllabic prepositional phrase (e.g., *der* vs. *einer* or *von der*; Kentner, 2018).

While rhythmic alternation on the word level was also dealt with by Chomsky and Halle (1968, p. 114–117) in The Sound Pattern of English, alternation preferences above the single word level found their formalization within Metrical Phonology (Liberman and Prince, 1977; Hayes, 1984; Selkirk, 1984). In their paper “On stress and linguistic rhythm” by Liberman and Prince (1977), which was a major contribution to Metrical Phonology, the authors highlight the relational nature of stress—the prominence of a syllable can always only be determined in relation to its surrounding syllables. They also provided a formal account for the so-called “rhythm-rule”—an instantiation of the Principle of Rhythmic Alternation. The rhythm rule operates in order to avoid stress clashes and to ensure an alternation of strong and weak beats, i.e., stressed and unstressed syllables, by shifting one of the clashing stresses to an earlier syllable. Consider a phrase like “15 women.” *Fifteen* in isolation has its primary stress on *-téen*, but when adjacent to a word with initial stress (*wómen*) primary stress is shifted to the first syllable onto *f*í*f* —resulting in *f*í*fteen wómen*—a phrase with an alternating stress pattern of strong and weak. Liberman and Prince (1977) refer to this (optional) process of stress retraction as *Iambic Reversal*.^4^

Forming rhythmically agreeable utterances is, as it happens, not only a matter of aesthetics as the term *eurhythmy* may suggest. Rhythm in language has a particular guiding function and makes speech understanding more effective: It supports the guidance of the listener's attention to relevant events in the utterance—on the one hand, different levels of boundaries and, on the other hand, prominently marked units relevant for sentence meaning (cf. Wagner, 2008; Kohler, 2009). Particular attention will be drawn to where the rhythmic beat is strongest, for example in stressed syllables (Pitt and Samuel, 1990; Quené and Port, 2003). An alternation of strong and weak syllables helps to highlight these points in time additionally, by making the stressed syllables in direct contrast to the adjacent weak syllables as salient as possible (cf. Schlüter, 2005).^5^

Several studies have demonstrated the effect on language production and processing if the expectation created by the rhythmic context are not met, even in silent reading (e.g., Magne et al., 2010; Breen and Clifton, 2011; Rothermich et al., 2012). There are only few studies, however, which consider how these tendencies interact with other prosodic functions above the word, such as the prosodic marking of information-structure categories. One of them (Kentner and Vasishth, 2016) investigated the German focus particle *auch* (“also”), which in the designed test sentences is unaccented when it occurs with object focus or VP focus, and accented when it occurs with subject focus. They found that the rhythmic context affected whether or not readers realized an accent on *auch*: When it occurs with subject focus they found more accent realizations when *auch* falls on a beat in a sequence of alternating strong and weak beats, and less accents when *auch* occurs off beat. This finding was mirrored in silent reading, where the “off-beat” *auch* resulted in increased reading times when it occurred with subject focus which required it to be accented (Kentner and Vasishth, 2016).

The rhythmic context may also be the driving factor in the findings by Riester and Piontek (2015), mentioned above. The authors suggest that pitch accents may be omitted, or shifted in order to avoid a pitch accent clash. It remains to be seen whether such an omission or shift will be a strategy that speakers also take in more marked contexts, such as contrastive focus.

Henrich et al. (2015), in fact, included information structure in an ERP experiment on the processing of rhythmic irregularities in German phrases. They investigated mini dialogues such as the following (emphasis in the original): “Was soll sie absagen? *WHAT is she supposed to cancel?*”—Sie soll den TERMIN absagen, wie besprochen. *She is supposed to cancel the APPOINTMENT, as discussed*.” They found that irregularities elicit weaker responses when the attention is shifted toward the preceding word (by contextually inducing a narrow focus, in this case on *Termin*). The target words themselves were not investigated under narrow focus marking.

The study on oral and silent reading by Kentner (2012a) centers around the ambiguous item *mehr*, which can have two readings with different prosodic realizations. The first one is the temporal reading of *nicht mehr* (in the sense of “no longer”). In this reading *mehr* is not expected to be accented. The other reading of *mehr* is comparative (in the sense of “more than”). In this usage *mehr* is required to be accented. He found that in unprepared oral reading, speakers more often did not produce the required accent on comparative *mehr* when the following word was stressed on the initial syllable (“...nicht MEHR nachweisen, als die Tatzeit”—*...not prove more than the date of the crime*.). However, with respect to focus marking, Kentner assumes that the main pitch accent in the phrase may not be altered by the desire for rhythmic alternation (cf. Kentner, 2012b).

So far we have talked about studies investigating the preference for an alternation of strong and weak beats. Rhythmic well-formedness is, however, not only about the regular occurrence of beats, but according to Jun (2012) refers to the temporal organization of speech by the regular occurrence of events in general. These events may be aural or visual, the acoustic medium may by timing, fundamental frequency or amplitude (Jun, 2012). Looking at fundamental frequency, this means that not only prominence and phrasing but also variation in pitch contributes to the perception of rhythm and rhythmic well-formedness, with an alternation of high and low being “more rhythmic” than sequences of level tones (Jun, 2012, 2014).^6^

In order to investigate whether alternation preferences on the tonal level play a role in German, Schauffler et al. (2015a) conducted a study on a corpus of German radio news recordings (Eckart et al., 2012). Motivated by the Principle of Rhythmic Alternation, they expected that speakers prefer the succession of alternating pitch accent types (e.g., L*H H*L) to the succession of non-alternating pitch accent types (e.g., H*L H*L). While a general tendency for alternation in the corpus was not found, it could be shown that the closer pitch accents are together, the more likely they alternate in type. This means that speakers indeed seem to prefer adjacent pitch accents to be different, and disprefer a succession of pitch accents with an identical contour. It was also found that the effect does not hold across intonation phrase boundaries (IP) or intermediate phrase boundaries (ip).

This study provides some evidence that there is a tendency for tonal alternation in German and that speakers try to avoid melodic clashes. How does this preference interact with the prosodic realization of information structure categories? Given that focus is not only realized by pitch accent placement, but is also often associated with a specific pitch accent type (see above), it is plausible that the rhythmic environments affect the prosodic structure in both—pitch accent placement and pitch accent type.

### 1.3. Research questions and overview of experiments

Summing up the premises—in German and English, information structure is marked by both pitch accent placement and pitch accent type, but there is variation. We also know that an alternation of both strong and weak beats and possibly F0 peaks and valleys is preferred. From these starting points the overall research question is derived, namely how does the preference for rhythmic alternation interact with the prosodic marking of focus—can alternation preferences account for some of the variation that we find in the prosodic marking of focus.

In order to elicit an interaction between focus marking and preferred vs. dispreferred rhythmic structures, all stimuli consisted of question-answer pairs eliciting adjacent corrective foci with different stress patterns either corresponding to alternation preferences or not (e.g., *g*í*rls rómance novels* vs. *g*í*rls advénture books*). Additional conditions tested the realization of the same sentences but with only one focus (either the first or the second noun phrase). Experiment 1 investigates double-focus sentences in German with German speakers. Experiment 2 replicates this experiment in English with English speakers. Experiment 3 investigates the status of the rhythm rule under corrective focus marking in English, also using double-focus constructions.

All experiments are oral reading experiments.

## 2. Experiment 1: alternation and focus marking in German

Experiment 1 investigates pitch accent placement and pitch accent type in German sentences with adjacent foci.

### 2.1. Participants

Sixteen (5 men, 11 women) German native speakers, recruited at the University of Stuttgart, participated in the experiment. Their mean age was 27.25 years (range: 19–33) and none of them had known speech or reading disorders. All participants were naïve as to the purpose of the experiment. They were paid for their participation.

### 2.2. Material

The stimuli were constructed as question-answer pairs that were designed in such a way that two noun phrases (NPs) introduced in the question needed to be corrected in the answer. To investigate influences of the rhythmic environment on the prosodic realization of the double-focus constructions, two conditions were designed: one eliciting pitch accents on successive syllables, which therefore directly follow each other (**clash** condition) and one condition in which the potential pitch accents are separated by an unaccented syllable (**no clash** condition). Examples are given in (3), lexically stressed syllables are underlined, small capital letters indicate focus expected to be marked by pitch accent.

(3) Hat Melli gesagt, dass Tobi das Schlagzeug Schülerinnen gegeben hat?
*Did Melli say that Tobi has given the drums to pupils?*
*Nein, sie hat gesagt, dass Tobi...*
*No, she said that Tobi..*.

**clash.**   ... *das [Kla*vier*]*_*NP*1_ *[*Leh*rerinnen]*_*NP*2_ *gegeben hat*.
     the piano    teachers   given  has
  *   ... has given the piano to teachers*.
**no clash**   ... *das [Kla*VIER*]*_*NP*1_  *[Stu*DEN*tinnen]*_*NP*2_ *gegeben hat*.
  the piano   students   given has
*... has given the piano to students*.

Two control conditions were added to each sentence type, in order to investigate the realizations of single-focus constructions under a corrective context. The control conditions also allow us to get an idea whether the participants generally processed the context question and understood the task. In condition *F1* only the first NP (NP1) was corrected while in condition *F2* only the second NP (NP2) was corrected [see examples in (4); *F1*=focus on NP1; *F2*=focus on NP2].

(4) Hat Melli gesagt, dass Tobi das Schlagzeug Schülerinnen gegeben hat?
Did Melli say that Tobi has given the drums to pupils?
Nein, sie hat gesagt, dass Tobi...
*No, she said that Tobi..*.

**F1.**   ... *das [Kla*VIER*]*_*NP*1_ *[Schülerinnen]*_*NP*2_ *gegeben hat*.
  the piano pupils given has
*... has given the piano to pupils*.
**F2.**   ... *das [Schlagzeug]*_*NP*1_ *[LEH*rerinnen]*_*NP*2_ gegeben hat*.
  the drums students given has
*... has given the drums to teachers*.

Since phrase length matters in the distribution and possibly choice of accents (cf. Ladd, 2008; Wang and Féry, 2018), the stimuli were controlled for number of syllables (8 words and 13 syllables starting from the embedded clause). In the double-focus conditions, the first focused word (in NP1) was always a disyllabic iamb, therefore carrying the lexical stress on the final syllable. NP2 always had four syllables with lexical stress on the initial syllable in the *clash* condition and lexical stress on the second syllable in the *no-clash* condition.

In order to avoid a segmental influence on the tonal marking, which would be especially expected for stops, there were only continuants in the coda of NP1 and no voiceless stops in the onset of NP2.

Additionally, all “contrasting pairs” (the group of NP1s and the group of NP2s) were controlled for word form frequency which were taken from the Leipzig Wortschatz corpus (Quasthoff and Richter, 2005).^7^

### 2.3. Procedure

Twenty sentences per condition (*clash, no clash, F1, F2*) were distributed over four lists using a Latin Square Design so that each participant read only one answer per context question. The experimental sentences in each list were pseudo-randomized for each participant so that the first three mini-dialogues were fillers and that sentences of the same condition were not successive. One list contained 20 experimental sentences and 40 filler sentences. The filler sentences did not involve double-focus constructions, but were dialogues of varying type, some involving one corrective focus, others no contrastive focus at all. The context questions of each question-answer pair had been previously recorded spoken by a female German native speaker who was instructed to read the questions in a neutral and natural way. The recordings took place in a sound attenuated chamber. The mini-dialogues (both question and answer) were presented on a screen, preceded by instructions and a context story introducing a hearing-impaired aunt in a family gathering. The story was designed to make the question-answer pairs more plausible.

It has been found that the reading modality influences accent distribution (Kentner, 2012a; Tilsen, 2012). In particular, effects of rhythmic repair strategies were predominantly found in prepared speech (Tilsen, 2012). Therefore, participants were instructed to first silently read the dialogue, listen to the context question and then produce the answer. This means they were prepared for their answer sentence. The speakers controlled the appearance of each new dialogue themselves by pressing a key on a keyboard. They were instructed to repeat their productions in case of misreadings. The instructions were also given verbally. This resulted in 320 produced answers.

### 2.4. Analyses and results

Three recordings were excluded from the statistical analysis because they either included major hesitations or repetitions (2), or they were realized with a different stress pattern than intended (‘*Beton* instead of *Be‘ton*, which is found in some German dialects), so that 317 recordings were statistically analyzed.

#### 2.4.1. Analysis of pitch accent placement

The collected recordings were first analyzed with respect to pitch accent placement in order to answer the first research question, namely do alternation preferences affect pitch accent placement? More specifically, are both focus accents realized in a clash environment, that is when the pitch accent carrying syllables follow each other, or are information-structurally required pitch accents omitted when their realization would lead to a clash?^8^ More precisely, are both focus accents realized in a clash environment, i.e., when the pitch accent-bearing syllables follow each other, or are information-structurally necessary pitch accents omitted if their realization would lead to a clash?

The following analysis is based on the judgements of three annotators. All recordings were played in random order to three annotators, who are trained in annotating intonation, using the Demo-window from praat (Boersma, 2001). All annotators have German as their L1. Two annotators are prosodically trained, but unaware as to the purpose of the experiment or the nature of the different conditions. One annotator was the author. Since prominence judgements are also highly biased by the listeners' expectation (e.g., Wagner, 2005), the answer sentences were played without the respective context question in order to gain a semantically (and theoretically) unbiased judgement. The annotators were asked to click on the word or words which sounded prominent to them from the determiner onward. Each recording was played three times in the course of the annotation experiment. This resulted in a total of 957 recordings that were annotated [one of the three recordings which were removed from the statistical analysis (see above) was already excluded at this point due to heavy hesitations]. The annotators were able to replay each recording up to 10 times before a decision was required.

A constituent was considered to be prominent when it was perceived as such at least four times, which means that more than one annotator perceived the prominence.^9^ This means that for each constituent there was one prominent value (1 or 0) in the end.

##### 2.4.1.1. Inter-annotator and intra-annotator agreement

Fleiss' Kappa (Fleiss, 1971) was calculated on the agreement on the prominence pattern (prominences on both objects, on NP1 or on NP2) given for each recording by one annotator compared to the other two annotations of the same recording by the same annotator (intra-annotator agreement) and to the annotations of this recording by the other annotators (inter-annotator agreement).

The intra-annotator agreements were κ=0.86 for annotator one, κ=0.78 for annotator two and κ=0.85 for annotator three. This means that all annotators had substantial to almost perfect agreement within their own ratings (cf. Landis and Koch, 1977). The inter-annotator agreements for the prominence judgement task was κ=0.77, which means that annotators agreed substantially on the prominence patterns of the recordings as played to them.^10^

##### 2.4.1.2. Results

All statistical analyses in the following sections were performed in R 3.5.0 (R Core Team, 2018), using the function glmer with the package lme4 (Bates et al., 2013). For data processing, and visualization the R packages “tidyverse” (Wickham, 2017), “plyr” (Wickham, 2011), “ggplot2” (Wickham, 2009), and “jtools” (Long, 2018) were used. An alpha level of 0.05 was used for all statistical tests.

A generalized linear mixed model using the logit link function was performed with the number of prominences (binomial: one or two) as dependent variable. As fixed factor *condition* (*clash, no clash, F1, F2*) was included, and as random effects intercepts for *subject*, and for *item*. An overall effect for condition was tested for significance by using likelihood ratio tests comparing the model including *condition* to the model without it (cf. Baayen, 2008; Baayen et al., 2008; Winter, 2013). The model with an AIC value (Akaike's information criterion) of at least two points smaller was considered the better model (cf. Burnham and Anderson, 2002) when its *p*-value was < 0.05.

**Figure 1:**
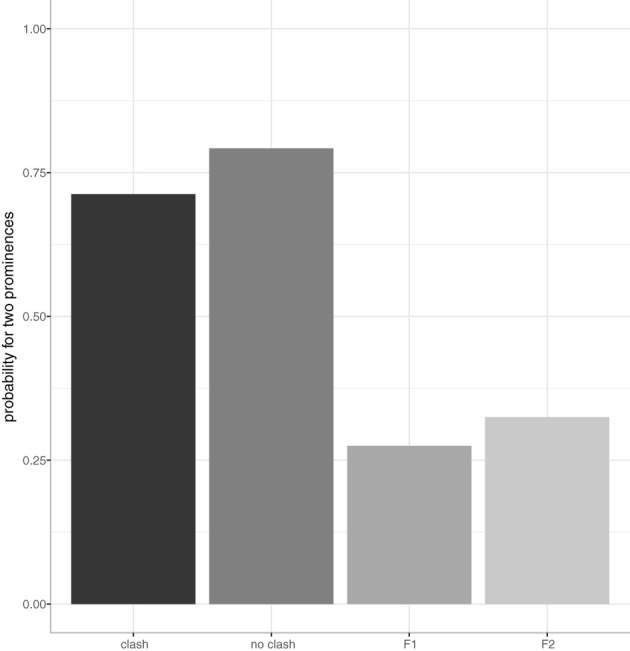
Number of prominences by condition (x-axis) in German data. The probability for two prominences (on both foci) is on the y-axis.

There is an overall effect of condition as determined by the likelihood ratio test [χ ${}_{\left(1\right)}^{2}$ = 75.70, *p* < 0.0001]. Figure 1 shows the results for the number of prominences as annotated by condition. On the x-axis we see the four experimental conditions and on the y-axis the probability for two prominences is given.

Looking at the control conditions *F1* and *F2*, where the answer sentences have only one corrective focus, we can see that single-focus sentences are mostly produced with only one prominence, as expected. These are significantly different from the double focus condition *clash* (27.5% for *F1*, β = −2.17, SE = 0.39, *p* < 0.0001; and 32.5% for *F2*, β = −1.90, SE = 0.38, *p* < 0.0001).

In double-focus sentences (conditions *clash* and *no clash*), speakers mostly produced a prominence on **both** focused constituents, namely in 71% of *clash* and in 79% of *no clash* sentences. The difference between these two conditions does not reach significance (β = 0.45, SE = 0.40, *p* = 0.26).

When only one prominence was annotated, the prominence was perceived almost always on the second NP in both clash and no clash conditions. This realization conforms with an all-new sentence where the nuclear accent would fall on the rightmost argument of the verb (NP2).

#### 2.4.2. Analysis of pitch accent type

In order to understand what kind of pitch accents speakers chose and whether the rhythmic environment affects the choice of pitch accent type, all double-focus instances with two realized prominences were annotated with respect to pitch accent type.

All double-focus sentences that were realized with two pitch accents according to the first annotation task, were annotated with respect to pitch accent type and phrase boundaries using GToBI(S) (Mayer, 1995).

The following labels were used for the annotation of pitch accent type:

1. H*L: a high tonal target on the accented syllable followed by a fall on the same or on the post-accented syllable. This label also includes its downstepped version, that is when the tonal target on NP2 is high but lower than the tonal target of NP1 (!H*L).
2. H* a high tonal target on the accented syllable without a fall, but here the contour stays high until the next pitch accent's high tonal target (linked leveltone).
3. L*H a low tonal target on the accented syllable followed by a rise on the same syllable or on the post-accented syllable. This label also includes L*, that is a rising accent whose H-tone is reached with the next pitch accent.

In cases of uncertainty the data was played to a phonetician trained in the annotation of intonation and discussed until a decision was reached. There was one case in which a pitch accent on the first NP was not perceived even though there were at least four out of nine judgements in favor of a prominence. Since this was a single case, this instance was disregarded from all further graphical and statistical investigation in order to improve readability.^11^

##### 2.4.2.1. Results

NP2 was always realized with a fall (H*L). The different realizations of the first focus will be referred to by looking at the whole contour encompassing NP1 and NP2. The following three realizations were identified:

1. **fall-fall pattern:** The first focus accent is realized with an H*L accent, this means that both foci were realized with a falling contour (this contour corresponds to the two-peak pattern described by Wang and Féry, 2018).
2. **high-fall pattern:** The first focus is realized with an H* accent, that is with raised F_0_, but there is a no low trail tone, that is no F_0_ lowering, between the two high targets (H* on NP1 followed by H*L on NP2).
3. **rise-fall pattern:** The first focus is realized with an L*H, that is a rising contour.

**Figure 2:**
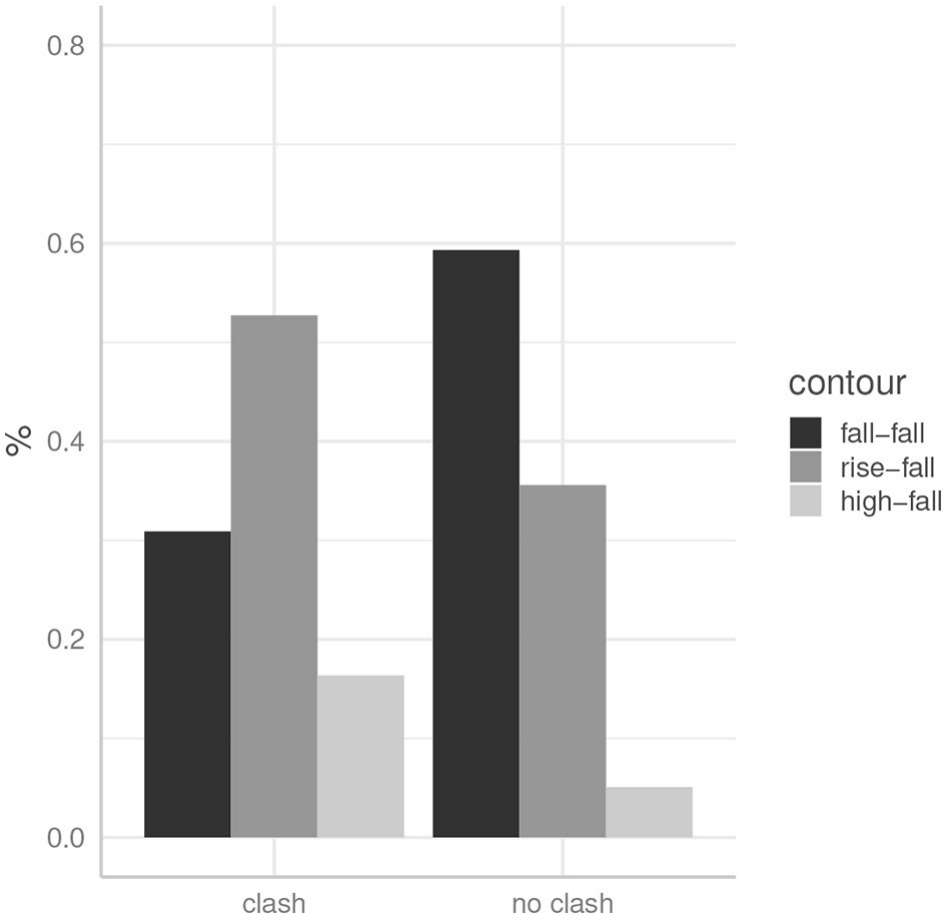
The distribution of pitch accent types across conditions in German data.

Figure 2 shows the distribution of pitch accent types across the two double-focus conditions. We can see that the contour expected for two successive corrective foci from an information-structural view, namely the fall-fall pattern (H*L on NP1 and H*L on NP2), is the preferred contour in condition *no clash*: when speakers produced two prominences, they used this contour in about 58% of the *no clash* sentences. In the rhythmically dispreferred condition *clash*, however, the preferred contour seems to be L*H on NP1, that is a rise-fall with a rising pitch accent on the first focus. Whenever speakers produced two prominences, they used this contour in 54% of the *clash* sentences. The rise-fall was, however, also quite frequently used in the *no clash* condition (36%) suggesting that this is an acceptable production also in rhythmically well-formed sentences.

In order to statistically test whether these differences are significant, a generalized linear mixed model was conducted using the logit link function. Since it looks like condition *clash* decreases the occurrence of fall-fall patterns and increases the occurrence of rise-falls and high-falls, the presence of fall-fall was used as dependent variable (binomial). As fixed factor condition (*clash* and *no clash*) was included, and as random factors an intercept for item and for subject. A model comparison using a likelihood ratio test comparing the final model to the null-model (without the fixed factor *condition*) revealed that there is an effect of condition [$\chi_{\left(1\right)}^{2}$ = 13.42, *p* < 0.001]. There are significantly more fall-fall realizations in condition *no clash* (β = 1.70, SE = 0.50, *p* < 0.001) compared to condition *clash*.

### 2.5. Summary and intermediate discussion

The aim of this experiment was to investigate whether a preference for alternation affects the prosodic realization of corrective focus. The presented analyses followed two research questions, namely first, whether alternation preferences affect pitch accent placement, more specifically, whether information-structurally required pitch accents are omitted in rhythmic clash contexts, and second, whether alternation preferences affect speakers' choice of pitch accent type used for focus marking.

The following will summarize and discuss the results in light of these research questions.

In most of the cases focus was perceived to be realized with a prominence in both single focus conditions as well as in double-focus conditions. This means that in the data at hand an information-structurally required pitch accent was not perceived in about 28% of *clash* sentences and in about 21% of *no clash* sentences. The difference between the two environments did not reach significance.

It is conceivable that the requirement to prosodically mark focus with a pitch accent is too strong in German to be easily overridden by rhythmic preferences. This is in line with results by Henrich et al. (2015) who showed in an ERP study that rhythmic irregularities are more likely to be tolerated when attention was guided toward a preceding context-induced narrow focus. A corrective focus, being directly contrasted to an alternative given in a question, such as in the current experiment, certainly is quite on the far end of a scale of emphasis given to information-structural categories. It remains to be seen in future work whether less emphatic information-structure categories are equally stable in rhythmically dispreferred structures.

Given the consideration that a corrective focus is typically realized with an emphatic pitch accent, the number of pitch accent omissions is relatively high in the condition that should be more in accordance with rhythmic preferences (*no clash*). One unaccented syllable may therefore not be enough to comply with the preference of some speakers for an alternation of weak and strong.

On the other hand, with about 28% of double-accent productions in *F1* and 33% in *F2* these numbers are slightly higher than expected. A higher number of prominent NP1 in condition F2, where NP1 constitutes given material, is less surprising since prefocal rising accents on given material are not uncommon (Baumann, 2006; Baumann and Grice, 2006; Féry and Kügler, 2008; Schweitzer et al., 2009). This does not apply to the instances in condition F1 with a post-focal accent on the given NP2, though. Contrary to the study in Schauffler et al. (2015b), all participants were instructed to read through the dialogue before producing the answer sentence. The preparation may have led to a more pronounced articulation which possibly resulted in the production of more pitch accents than in unprepared speech.

While the rhythmic environment did not significantly affect pitch accent placement, it was found that speakers preferred different pitch accent types in rhythmically preferred (*no clash*) vs. rhythmically dispreferred (*clash*) sentences. The pitch accent type typically associated with contrastiveness, a fall, was more often found in the rhythmically regular condition, while a rising contour was preferred in clash environments. This means that the rhythmic environment affected how the first focus was prosodically realized. Finding the rise also quite frequently in regular contexts, it was confirmed that double-focus sentences are not merely composed of two single foci but that the first focus can be realized with different pitch accent types. Wang and Féry (2018) found that the rise is the preferred contour in short sentences. In the current experiment sentence length was controlled and both conditions can be considered short sentences in the sense that there was no, or only one syllable between the two focus constituents. This may explain the frequent use (about 36%) of rising contours in regular contexts.

The rise-fall contour, however, is more often used in clash contexts, suggesting that an alternating contour is preferable in cases of pitch accent clash. This is in line with the findings of the corpus experiment by Schauffler et al. (2015a), that pitch accents are more likely to alternate in type when they are close together. The current experiment showed that this alternation preference has an influence on the marking of corrective focus.^12^

One of the contours that was less frequently used (more often in condition clash with 18.5 and 6.8% in condition no clash) was the high-fall pattern, that is a high pitch accent H* on the first focus followed by a falling H*L pitch accent on the second focus. The underlying pitch accent, according to GToBI(S), is an H*L whose low trail tone is linked to the following pitch accent, that is the fall only happens later with the next falling accent (cf. Féry, 1993; Mayer, 1995). According to this reasoning the high-fall pattern should be a phonetic variant of the fall-fall contour. It needs to be tested whether this contour then is equally accepted in the context of corrective focus, since the pitch accent type typically associated with corrective foci is a fall with raised F_0_ and post-focal F_0_ compression (Grabe, 1998; Baumann and Grice, 2006; Braun, 2006; Féry and Kügler, 2008). However, the falling part is missing in a linked H* accent.^13^

In an experiment on Dutch, Chen (2012) found that the amount of sonorant material affected whether speakers used H*L or H*(L), irrespective of information structural categories (topic or focus). While speakers used an H*L accent when there was enough sonorant segments to realize the bitonal contour, they used a monotonal H* when less sonorant material was present. The author concludes from this finding that the distinction between the two accent types is rather phonetic in nature. In the current experiment the H* variant was found more often in the clashing context, that is when there was no material between the two accented syllables which seems to support the assumption that it is a phonetic variant. However, only one syllable between the two accents does not seem to be enough material in order to result in such a low number of high-fall patterns in the *no clash* condition. In fact, if H* and H*L were merely phonetic variants of the same contrastive pitch accent we would have expected a greater number of high-fall contours across conditions. While intuitively post-focal lowering seems essential in the interpretation of contrastive focus, further perception studies including multiple focus sentences are needed in order to investigate the role of the fall in focus accents in German.

From the main findings of this first experiment it can be concluded that pitch accents can influence each other inherently according to alternation preferences, and that these preferences can influence the prosodic realization of focus in terms of pitch accent type. It seems like in cases of pitch accent clash, i.e., in cases where an alternation of strong and weak is not possible, speakers may revert to an alternation of pitch accent types in order to better integrate the clashing accents.

## 3. Experiment 2: alternation and focus marking in English

The following experiment is set to investigate whether in English, sharing both the preference for rhythmic alternation as well as the prosodic marking of focus, speakers follow a similar strategy when confronted with rhythmically dispreferred structures in focus environments. To this end, Experiment 2 is a close replication of Experiment 1 with respect to material and set up.

### 3.1. Participants

Sixteen (4 men, 12 women) speakers were recorded at the Western Sydney University in Australia in a quiet room. Their mean age was 27.38 (range: 19–36). None of the participants had known speech or reading disorders. All participants were naïve as to the purpose of the experiment and were paid for their participation.

### 3.2. Material

Again, question-answer pairs were designed to elicit two adjacent pitch accents to prosodically mark the corrective foci: on directly adjacent syllables in the rhythmically dispreferred condition (*clash*) and with an unaccented syllable between the potential pitch accents in the rhythmically preferred condition (*no clash*). Examples are given in (5); lexically stressed syllables are underlined, small capital letters indicate focus expected to be marked by pitch accent. Square brackets indicate the target NPs.

(5) Did Carl say that Clara gave the boys horror stories to read?
  No, he said that...

**clash.**   ... *she  gave  [the*  GIRLS*]*_*NP*1_  *[*RO*mance  novels]*_*NP*2_.
**no clash.**   ... *she  gave  [the  *GIRLS*]*_*NP*1_  *[ad*VEN*ture  books]*_*NP*2_.

Also the English material contained two control conditions: In condition *F1* only the first NP was corrected while in condition *F2* only the second NP was corrected (see examples in 6).

(6) Did Carl say that Clara gave the boys horror stories to read?
  No, he said that...

**F1.**   ... *she  gave  [the*  GIRLS*]*_*NP*1_  *[horror stories]*_*NP*2_.
**F2.**   ... *she  gave  [the  boys]*_*NP*1_  *[*RO*mance novels]*_*NP*2_.

Like in the German stimuli, the first object was a disyllabic iamb—in 55% a monosyllabic noun with a preceding article (e.g., *the girls*) and in 45% a disyllabic proper name with final stress (e.g., *Estelle*), except for condition F2, where the disyllabic proper name was repeated from the context question and was therefore initially stressed (e.g., *Did Ryan say that he lost Rory's master key?—No, he said that he lost Rory's access code*). The second NP had the lexical stress on the first syllable in *clash, F1*, and *F2* and on the second in the *no-clash* condition. The number of syllables of the second NP ranged from two to seven but was kept constant within one item [that is across the four conditions with the same lexical material, as in (5) and (6)] except for few cases where there was a difference of no more than one syllable between the conditions. This means that there was no substantial difference in length between the conditions.^14^

Additionally, all target constituents were controlled for word form frequency (or compound frequency, respectively) which were taken from the wackipediaEN corpus (Baroni et al., 2009). Frequencies were matched within item and position: words in first NP position are comparably frequent per item, and words in second NP position are comparably frequent per item.

In order to avoid a segmental influence on the intonational marking, which would be especially expected for stops, there were only continuants in the coda of the first NP and no stops in the onset of the second NP. Note that compared to the German stimuli for the production study, the subject in the answer sentences was exchanged with a pronoun in the English material to reduce the complexity of the sentences.

All English material was reviewed by one American English speaker and by one Australian English speaker in terms of grammaticality, acceptability and plausibility. Additionally, the material was tested in a pilot study.

### 3.3. Procedure

Twenty sentences per condition (*clash, no clash, F1, F2*) were distributed over four lists using a Latin Square Design in the same ways as in experiment 1 (see Section 2.3). The stimuli of Experiment 3 presented in the following Section (4) were integrated in the current experiment, so that one list contained 20 experimental sentences of the “clash”-experiment (Experiment 2), 20 sentences of Experiment 3 which will be referred to as “shift”-experiment, and 100 filler sentences. The context questions of each question-answer pair had been previously recorded spoken by an Australian English native speaker. The recordings took place in a quiet room at the Western Sydney University. As in Experiment 1 (on German), a story with a hearing-impaired aunt was used to make the dialogues more plausible. The aunt's question was the pre-recorded context-question and the participants were asked to click on a symbol which triggered playing of the question on loudspeakers.

Participants were instructed to first silently read the dialogue, listen to the context question by clicking on the loudspeaker and then to produce the answer. This means they were prepared for their answer sentence.

### 3.4. Analyses and results

Procedure and analyses follow the same steps as for Experiment 1.

#### 3.4.1. Analysis of pitch accent placement

All recordings were played to three annotators using the Demo-window from praat (Boersma, 2001). One of the annotators was an American English native speaker and the other two annotators were German native speakers proficient in English (one of which was the author). All annotators were prosodically trained. The answers were played without the respective context question and in randomized order. Each recording was played three times; recordings of Experiment 2 and 3 were presented in one annotation task. The task was divided into 12 blocks à 160 sentences per group. This resulted in 1917 annotations, including 957 for the current Experiment 2 (the “clash-experiment”). Annotators could take breaks between blocks, and each recording could be replayed up to ten times before a decision was required.

##### 3.4.1.1. Inter-annotator and intra-annotator agreement

The intra-annotator agreements were κ = 0.81 for annotator one, κ = 0.69 for annotator two and κ=0.76 for annotator three. This means that all annotators had substantial to almost perfect agreement within their own ratings (cf. Landis and Koch, 1977). The inter-annotator agreements for the prominence annotation task was κ=0.6, which means that annotators agreed moderately on the prominence patterns of the recordings as played to them.

##### 3.4.1.2. Results

A generalized linear mixed model using the logit link function was performed with the number of pitch accents as dependent variable (binomial: one or two). As fixed factor, *condition* (*clash, no clash, F1, F2*) was included, and as random effects, intercepts for *subject* and *item*.

There is an overall effect of condition as determined by the likelihood ratio test comparing the model including *condition* to the model without it (cf. Baayen, 2008; Baayen et al., 2008; Winter, 2013) [$\chi_{\left(1\right)}^{2}$ = 34.33, *p* < 0.0001].

**Figure 3:**
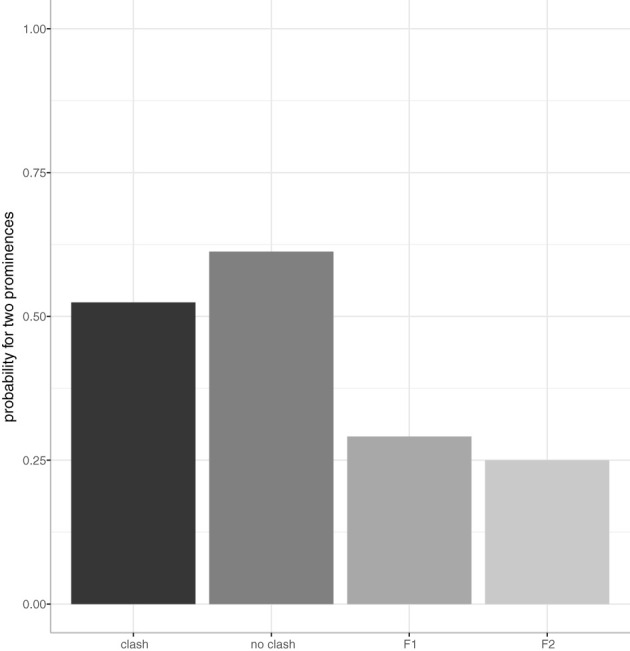
Number of prominences by condition (x-axis) in English data. The probability for two prominences (on both foci) is on the y-axis.

Figure 3 shows the results for the number of prominences as annotated by condition. On the x-axis we see the four experimental conditions and on the y-axis the probability for two prominences is given. Let us first look at the single-focus conditions F1 and F2, where the answer sentences have only one contrastive focus: We see that single-focus sentences only rarely are produced with two prominences which means that they are mostly produced with only one pitch accent, as expected, namely in 71% of *F1* (β = −1.13, SE = 0.36, *p* < 0.01 compared to *clash*), and in 75% of *F2* (β= −1.38, SE = 0.38, *p* < 0.001 compared to clash).

Looking at the two double-focus conditions, speakers realized two prominences in the majority of cases—slightly more often in the no clash condition (61%) compared to the clash condition (52%), but this difference is statistically not significant (β = 0.40, SE = 0.34, *p* = 0.24), this means that the rhythmic manipulation did not affect pitch accent placement. But note that there are frequent omissions of pitch accents, in 48 and 39%, respectively. Whenever there was only one pitch accent realized in the double-focus sentences, it was almost always realized on the second focus.

#### 3.4.2. Analysis of pitch accent type

The analysis presented in this section has a closer look at the realized pitch accents as to their shape.

A subset of the data comprising the two double-focus conditions *clash* and *no clash* was annotated by the author whenever at least 4 out of 9 annotations marked both NPs as prominent (that is all cases where both foci were considered to be pitch accented, see Section 3.4.1). Thus, 43 sentences of condition *clash* and 51 sentences of condition *no clash* were labeled with respect to pitch accent type and phrase boundary.

In order to be able to compare the realizations by English speakers to the realizations by the German speakers in this study the same category labels were used as in Experiment 1. These labels stand somewhat aside the mainstream labeling conventions for English mainly serving the purpose of comparability. Grabe (1998) suggests a similar inventory in comparing German and English intonation. She bases her annotation on a modified version of Gussenhoven's model for British English and Féry's model for German. In both languages she transcribes rises with L*+H and falls with H*+L. The difference to the annotation here is that in the study at hand also H* is used for a high pitch accent that is not followed by a fall, but linked to the next pitch accented syllable. The different categories are based on whether the accented syllable is perceived as high (H*) or low (L*).^15^ See Section 2.4.2 for an introduction of the annotated pitch accent types used and for the labels used to refer to the contour across the two foci.

There were two cases in which no pitch accent was perceived on the second NP even though there were four out of nine judgements in favor of a prominence. Since this is such a low number, these two instances were disregarded from all further graphical and statistical investigation. In the rest of the recordings, speakers always realized an H*L on the second focus which is expected given that the pitch accent on NP2 is the last accent in a statement. In 10 cases (five of condition *clash* and five of condition *no clash*) a pitch accent on the first focus was not perceived even though the annotators annotated a prominence at least four out of nine times. Since the number is not trivial, these cases are included in the following descriptions and referred to as NONE.H*L (no accent on NP1 and H*L on NP2).

##### 3.4.2.1. Results

**Figure 4:**
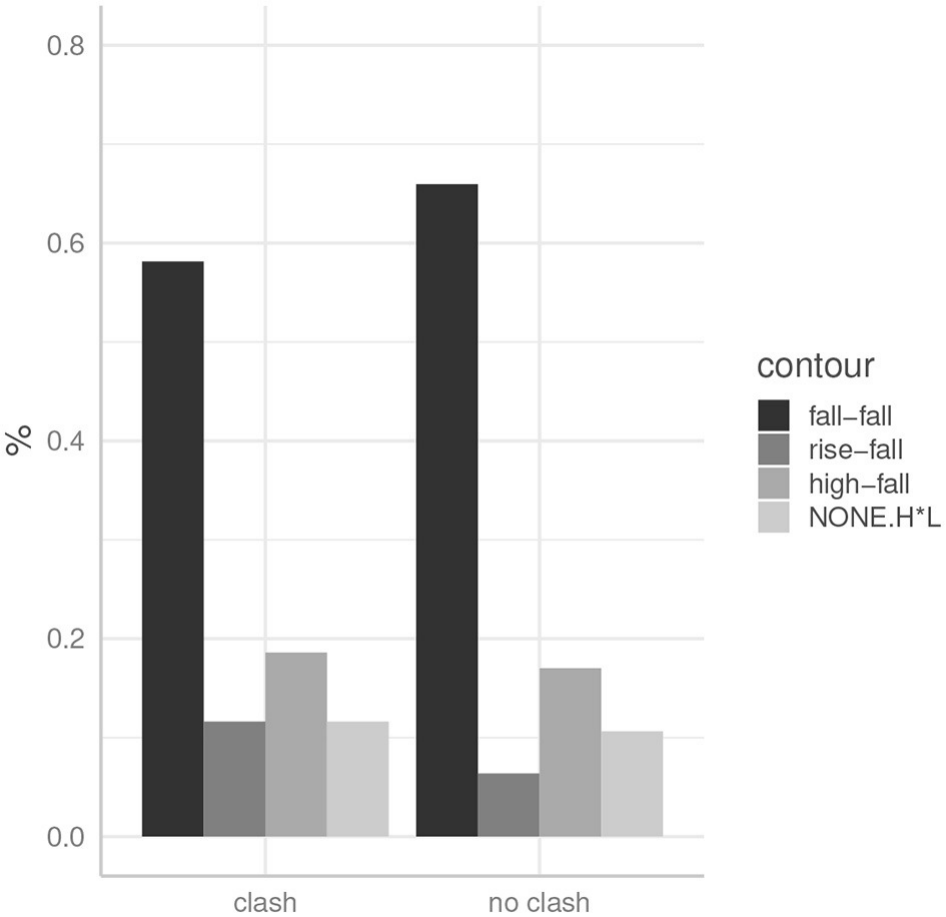
The distribution of pitch accent types across conditions in English data.

Figure 4 shows the distribution of contours across the two double-focus conditions. We can see that, contrary to the German data, speakers preferred the fall-fall pattern in both conditions, that is irrespective of the rhythmic environment. A generalized linear mixed model was conducted to examine the effect of *condition* (*clash* vs. *no clash*) on whether or not fall-fall was used, with *item* and *subject* specified as random intercepts. The model used the logit link function and a binomial distribution for the dependent variable. Model comparison using a likelihood ratio test indicated that including the factor *condition* does not improve model fit when this model is compared to the null-model without *condition* [$\chi_{\left(1\right)}^{2}$ = 0.57, *p* = 0.45]. Correspondingly, the simpler model is preferred.

### 3.5. Summary and intermediate discussion

The aim of this experiment was to elicit data that gives insight into how alternation preferences interact with prosodic focus marking in English. The results are going to be compared to the results found for German.

The research questions were the same for both languages, namely first, do alternation preferences affect pitch accent placement? And more specifically, are information-structurally required pitch accents omitted in rhythmic clash environments, given that the reduction of prominence is a strategy that has been found in the context of lexical stress clash (Horne, 1990; Grabe and Warren, 1995; Vogel et al., 1995)? And second, do alternation preferences affect speakers' choice of pitch accent type used for focus marking?

The results did not show an effect of rhythmic manipulation: the analysis did not reveal a difference in prominence distribution between the condition with a syllable between the two potential pitch accents compared to the condition with no material in between. With respect to pitch accent placement, this is in line with the German experiment where we did not see an effect of the rhythmic manipulation in the double-focus sentences. It was speculated there that the prosodic marking of a correction is too strong a constraint to be affected by alternation preferences, since mostly both foci were accented. What is striking in the English data, however, is the low number of realizations with two focus accents. Only 52% in the *clash* condition, and 61% in the *no clash* condition were realized with two prominences. This questions the explanation that the requirement to prosodically mark corrective focus is too strong to be bended by rhythmic constraints in English. If this were the case then we would expect a higher number of double-accent productions in both double-focus conditions.

So why was the first pitch accent often omitted in both *clash* and *no clash* contexts? There is no indication that this is an effect of having two foci in general—studies investigating multiple foci in English found that speakers reliably realize focus accents as expected. So the question is—do the missing pitch accents in both of these conditions possibly point toward an alternation effect after all? Possibly, for English speakers, one unaccented syllable between the two foci is not enough in order to dissolve the clash. In English, unstressed syllables are much more reduced than in German, with the vowel typically realized as a schwa (e.g., Delattre, 1969), so that there is a difference in quality between the English and German material between the two potential focus accents. This may entail that the condition *no clash* is still rhythmically dispreferred even though the focus accents are not directly adjacent on the lexical level, but only at a higher level in the prosodic hierarchy.^16^

In order to get a better understanding in this matter, all double-focus recordings where only one focus was annotated as being prominent were analyzed again by the author. It was found that in 39% of these cases (about evenly distributed across the two conditions) the given verb was pitch accented, as exemplified in (7).

(7) Did Carl say that Clara gave the boys horror stories to read?
  No, he said that she GAVE the girls ROmance novels.

It seems like the clash was resolved in some cases by shifting the prominence from the focused word *girls* to the given verb *gave*. Shifting prominence is something English speakers are used to do in rhythm rule contexts where stress is shifted to the left in order to avoid two successive stressed syllables (*thirtéen* and *th*í*rteen mén*). It may be the case that prominences on a higher level can also be shifted in order to avoid two directly adjacent pitch accents as has been suggested by Riester and Piontek (2015) (see above).^17^ The experiment presented in the next Section 4 will shed some light on this issue. There, the first focus in the double-focus conditions consists of stress-shift items (e.g., *thirteen*). This means a shift of stress to the initial syllable adds more material between the two foci. If this results in more double-accent realizations than in the present experiment this would hint at a rhythmic effect here in both conditions.

When interpreting the results from the annotation task for pitch accent placement, it has to be taken into account that there is a structural difference in some of the English stimuli compared to the German stimuli from Experiment 1. Specifically, in 55% of the test sentences the first focused word in the double-focus sentences was monosyllabic. This means that speakers had less material to realize a focus accent. This may have resulted in compressed accents that may not have been perceived as prominent (see Yu and Zahner-Ritter, 2018 on truncation and compression in Australian English and Southern German, which are the varieties considered here).

In those cases in which two prominences were perceived in the double-focus conditions, speakers mostly used the fall-fall pattern, that is each of the foci was realized with a fall. This is in line with previous findings by Eady et al. (1986), namely that the pitch accents with which English speakers in their experiment marked foci were falling in both single and double-focus sentences.

Even though the prosodic marking of single-focus is fairly similar across English and German, the two languages apparently differ when it comes to the prosodic marking of multiple foci in a sentence. While speakers of English seem to prefer to mark each focus with a fall regardless of whether another focused word follows, German speakers can choose between a high or a low tonal target on the first focus. This alternation of pitch accent types in clash environments is not available to English speakers. This may have direct consequences on the placement of pitch accents as discussed above. Not being able to adapt the pitch accent type to the rhythmic circumstances may consequently lead to more omissions of pitch accents altogether.

## 4. Experiment 3: alternation and focus marking in English rhythm rule contexts

Experiment 3 has a closer look at rhythmic repair strategies on the lexical stress level (i.e., rhythm rule contexts) and how this may interact with the prosodic marking of corrective focus.^18^

There are some intuitions and findings concerning corrective focus marking in rhythm rule contexts. According to Liberman and Prince (1977), stress shift will not take place when the word carries the nuclear accent of a phonological phrase. Beckman et al. (1990) found empirical evidence for this intuition in some instances where stress was not shifted when the target word was emphatically contrasted to some given alternative.

In a double-focus sentence, however, the phonological phrase may in fact carry two equally prominent accents, a context that has not been investigated with respect to stress shift. Experiment 3 therefore investigates the interplay of the rhythm rule with focus accent placement looking at different focus scenarios. It aims at answering the following questions: How does the rhythm rule operate under (corrective) focus marking? Does focus accent override the rhythm rule?

In addition to that, the design allows us to look at various other questions that are still debated. Previous studies on English have found that decreasing prominence of the primary stressed syllable (rather than increasing prominence on the secondary stressed syllable) is the dominant repair strategy in rhythm rule contexts (Horne, 1990; Grabe and Warren, 1995; Vogel et al., 1995). Since the marking of a corrective focus typically requires to increase prominence lending cues on the speaker side, it should become apparent whether stress is actually shifted since a mere reduction of prominence on the clashing syllable is not enough to arrive at an emphatic pitch accent.

The different conditions (see below) should also give insight into what constitutes a stress clash, that is under what circumstances is stress shift triggered (if at all under focus marking).

Eventually, Experiment 3 may shed light on findings from Experiment 2: English speakers often had produced only one prominence in double-focus sentences even when there was one unaccented syllable between the two potential focus accents. In Experiment 3, repair strategies are possible since there is a secondary stressed syllable to which prominence could be moved. The speakers' behavior in resolving clashes when stress shift is possible may give an explanation as to why speakers often omitted focus accents in Experiment 2.

### 4.1. Participants

The participants of this experiment were the very same as described in Section 3.1.

### 4.2. Material

The experimental stimuli were designed to elicit adjacent pitch accents via double-focus environments as has been described in the experiments above. Example (8) presents the four conditions for one item. Capital letters indicate focus expected to be marked by pitch accent, square brackets indicate focus. The first focus consisted of a word with secondary stress on the first, and main stress on the final syllable. The second focus had lexical stress on the first syllable in the *clash* condition, resulting in a stress clash on the lexical level, and on the second syllable in the *no clash* condition, i.e., with an unaccented syllable between the two potential pitch accents.

In addition, two single focus conditions were tested (*S-Foc* and *given*). Both conditions include a stress clash on the lexical level, but in *S-Foc* (= “single focus”) the target word is focused and the following word given; while in *given*, the target word is given and the following word focused.

The first focus, that is the target word possibly undergoing stress shift, was either an adjective (e.g., *unfair*), a *-teen* number (*fifteen*) or the first constituent in a compound (e.g., *infra-red microscopes*) with secondary stress on the first and primary stress on the final syllable.

It has been shown that more frequent words are more likely to undergo stress shift than less frequent words (Hammond, 1999). This is why also these stimuli were controlled for frequency as taken from the English corpora collection from Leipzig Wortschatz corpus (Quasthoff and Richter, 2005).

(8) Did Anna say that she met an Indian programmer?No, she said that ...

**clash.**   ... she met [a JapaNESE] [ARchitect].
**no clash.**   ... she met [a JapaNESE] [acCOUNtant].
**S-Foc.**   ... she met [a JapaNESE] programmer.
Did Anna say that she met a Japanese programmer?.
     No she said that ...
**given.**   ... she met a Japanese [ARchitect].

### 4.3. Procedure

Twenty sentences per condition (*clash, no clash, S-Foc, given*) were distributed over four lists using a Latin Square Design. The stimuli were elicited together with the stimuli from the previous experiment so that each list additionally contained the 20 sentences from that experiment and 100 filler sentences (see above).

After the dialogues, the participants read a list containing the target words in isolation interspersed with filler words with various stress patterns. Thus, 320 target sentences and 320 isolated words were recorded. Three sentences were excluded due to hesitations and verbal errors so that 317 sentences were analyzed.

#### 4.3.1. Stress shift annotation

Three phonetically trained listeners (two German speakers and one American English speaker) listened to the target words (removed from context) and the words produced in isolation, and indicated whether main stress was on the first or the last syllable by clicking on the respective syllable; each recording was presented twice in the course of the judgement task.

Intra- and inter-annotator agreement were determined using Fleiss' Kappa (Fleiss, 1971). For the L1 data, the intra-annotator agreements were κ = 0.86 for annotator one, κ = 0.76 for annotator two, and κ = 0.74 for annotator three. This means that All annotators had substantial to almost perfect agreement within their own ratings (κ = 0.86, κ = 0.76, κ = 0.74), inter-annotator agreement was κ = 0.49, which means that annotators agreed moderately (Landis and Koch, 1977).^19^

Stress was considered to be shifted when at least four out of the six listener judgments marked main stress on the first syllable. In 4.5% of cases neither *shift* nor *no shift* could be determined. Given the low number, these cases were removed from the statistical analysis when four out of six judgments agreed on equal prominence.

#### 4.3.2. Pitch-accent annotation

In order to understand the interaction of stress clash, stress shift and pitch accent placement, the three annotators judged whether they perceived the words involved in the rhythm rule as prominent or not. All answer sentences were played without the respective context question, and in randomized order.

This task was annotated together with the prominence annotation of Experiment 2, this means that the sentences of both experiments were presented in the same task in randomized order. Each recording was played three times in the course of the annotation task. Intra-annotator agreement was substantial to almost perfect (κ = 0.82 for annotator one, κ = 0.75 for annotator two, and κ = 0.75 for annotator three), and inter-annotator agreement was moderate (κ = 0.62).

### 4.4. Analyses and results

To investigate the relationship between stress shift and the five conditions (the four conditions of 8 plus the realization of the target word in isolation), a generalized linear mixed effects analysis using the logit link function was performed, with *stress shift* as the dependent variable. *Condition* was included as a fixed factor, and *subjects* and *items* as random intercepts.

**Figure 5:**
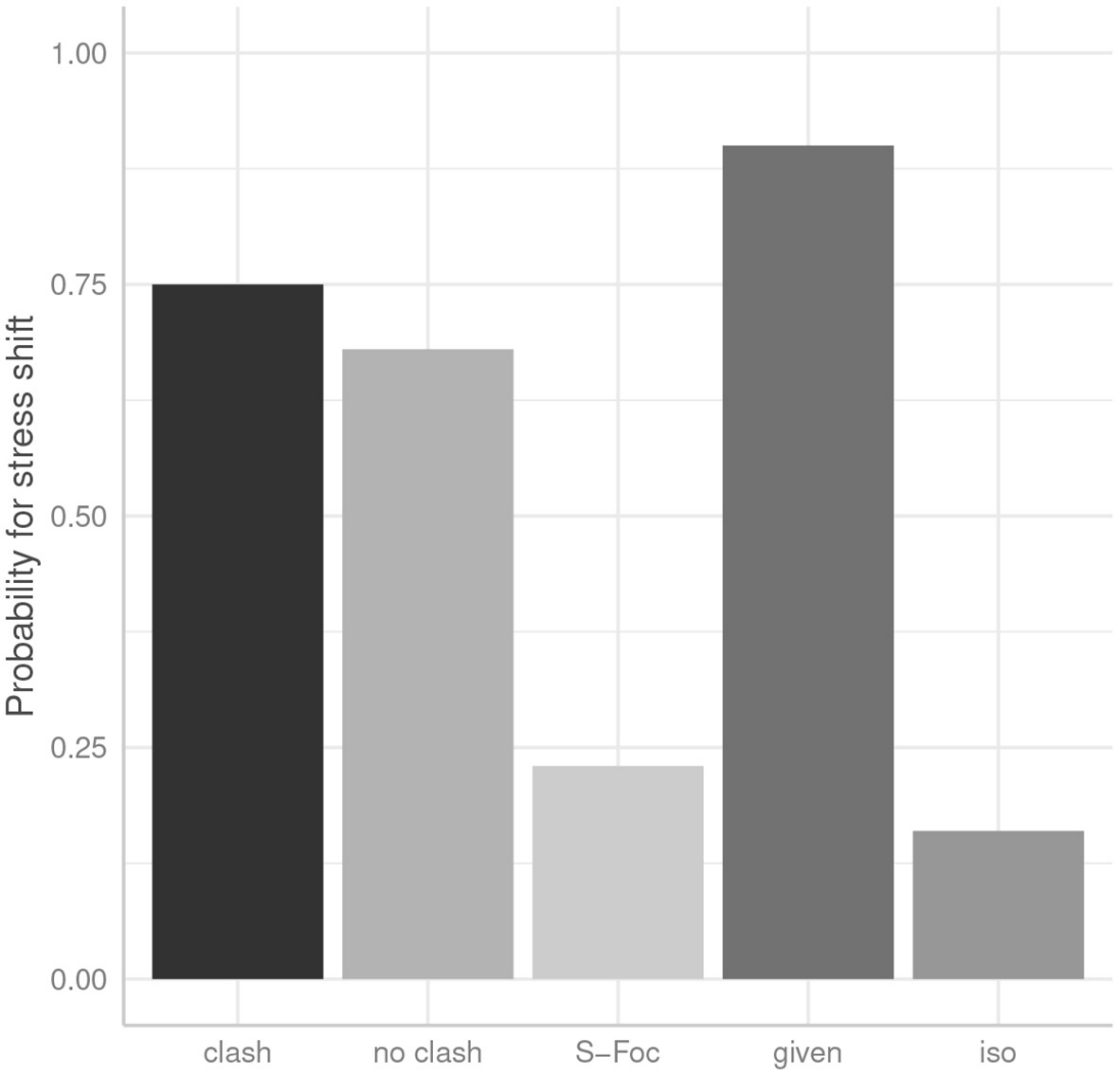
The probability for stress shift by condition in the English data.

There is an overall effect of condition as determined by the likelihood ratio test [$\chi_{\left(1\right)}^{2}$ = 270.32, *p* < 0.0001]. Figure 5 shows the probability of stress shift (y-axis) by the five different conditions (x-axis).

In condition *clash*, where two focus-marking pitch accents are required on directly adjacent syllables, stress was shifted in 75% of the cases. In condition *no clash*, stress was also shifted in the majority of cases (68%), even though the two foci were separated by one syllable in this condition, that is there was no stress clash on the lexical level. The two double-focus conditions were not significantly different (β = −0.42, SE = 0.38, *p* = 0.27).

Stress was shifted even more often (89%, β = 1.22, SE = 0.48, *p* < 0.05) when the target word was given and the following word focused (*given*).

Stress was **not** shifted in 77% of the cases when the target word was focused and the following word given (S-Foc) (β = −2.68, SE = 0.41, *p* < 0.0001), and in 84% of the cases when the word was produced in isolation (β= −3.19, SE = 0.35, *p* < 0.0001).

To investigate the correlation between stress shift and pitch accent placement, a second analysis was performed on a subset of the data comprising the two double-focus conditions (*clash* and *no clash*) using the presence of two pitch accents as dependent variable. An interaction between *condition* and *stress shift* was included as a fixed factor, and random intercepts for *subjects* and *items*. An overall effect of condition and the interaction term, respectively, was tested for significance by performing a likelihood ratio test comparing the model including the fixed factor to the model without it (cf. Baayen, 2008; Baayen et al., 2008; Winter, 2013). The model with an AIC value (Akaike's information criterion) of at least two points smaller was considered the better model (cf. Burnham and Anderson, 2002).

**Figure 6:**
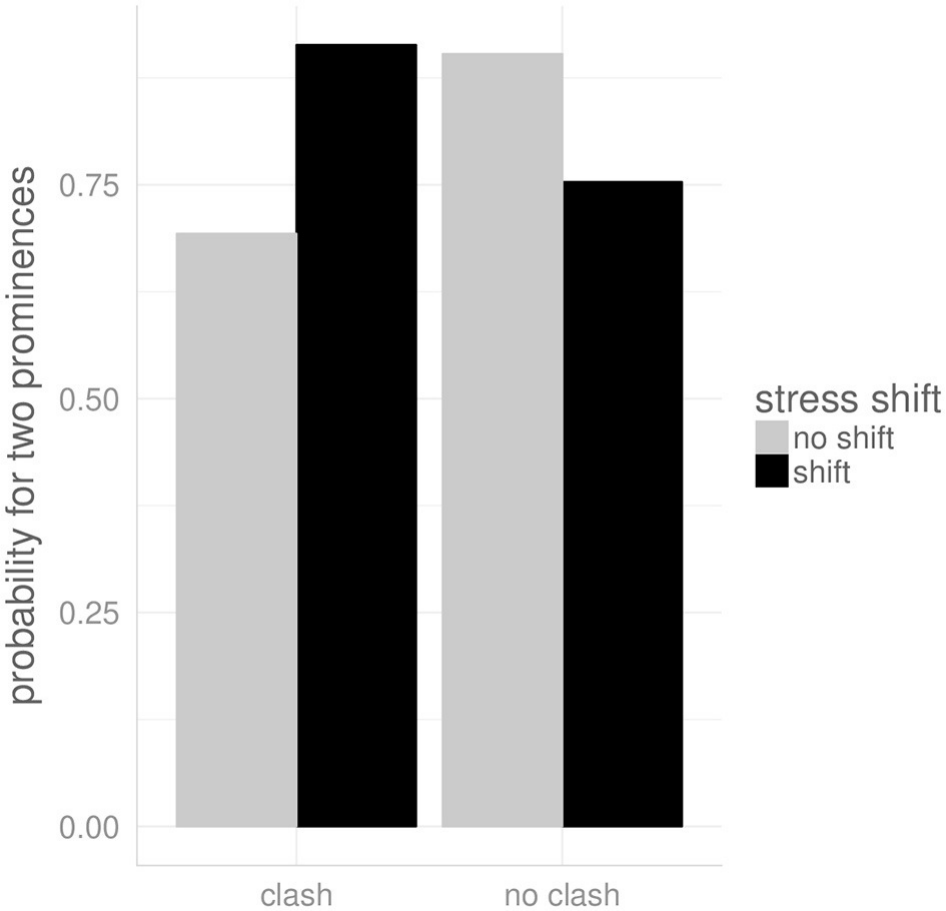
The propability for two prominences by condition and stress shift in the English data.

Figure 6 shows the probability for two prominences in the double-focus conditions. Including the interaction between stress shift and pitch accent placement significantly improves the model as determined by the likelihood ratio test [$\chi_{\left(1\right)}^{2}$ = 147.59, *p* < 0.05]. We found that while shifting stress by tendency increases the probability for the presence of two prominences from 69 to 91% (estimated) in condition *clash* (β = 1.54, SE = 0.81, *p* = 0.06), it decreases it in condition *no clash* from 90 to 75% (β = −2.66, SE = 1.09, *p* < 0.05).

### 4.5. Summary and intermediate discussion

This experiment aimed at getting insight into how and when the rhythm rule operates in sentences with varying focus structure. Specifically, the question was whether corrective focus overrides stress shift as has been previously assumed (see above). Comparing the three conditions in which the target word was focused (*clash, no clash, S-Foc*), it was found that English speakers hardly ever shift stress when the following word is given (*S-Foc*). The stress clash on the lexical level is not enough to trigger stress shift of the more prominent focused target word. When a second focus accent followed the target word, however, such as in the double-focus conditions *clash* and *no clash*, stress was shifted most of the time.

Concerning the first research question whether a corrective focus accent overrides the rhythm rule, it can therefore be specified that it does override the rhythm rule (so stress is not shifted) when it is the last accent in the phrase, and that it does not override the rhythm rule (so stress is shifted) when it is followed by another pitch-accented syllable as in the double-focus conditions. It seems that the second prominence needs indeed to be stronger (or equally strong) than the first prominence in order to trigger stress shift, as is also the case in *given*, where speakers almost always shifted the weaker stress to the left. While in *S-Foc*, the weaker prominence in the postnuclear word is not able to trigger the stronger prominence to shift (cf. Selkirk, 1984).

The fact that the annotators frequently perceived stress shift in the *given* condition suggests that the rhythm rule also operates in deaccented speech. This speaks against proposals that stress shift phenomena are caused by deleting or shifting a pitch accent (cf. Gussenhoven, 1991; Shattuck-Hufnagel, 1995).

Having a closer look at the double-focus conditions, it was found that English speakers often shift stress not only when corrective focus accents are located on directly adjacent syllables (as in condition *clash*), but also when the corrective focus accents are separated by one syllable (as in condition *no clash*), similar to findings by Vogel et al. (1995) and Beckman et al. (1990). This suggests that stress shift is not only triggered by directly adjacent lexical stresses on the syllable level, as implied by for example Henrich et al. (2014) or Vogel et al. (1995), but also by prominences of a higher level in the prosodic hierarchy as suggested by Standard Metrical Phonology (Liberman and Prince, 1977; Selkirk, 1984; Nespor and Vogel, 1986). This experiment could clarify that this includes pitch accents used for focus marking.

Since the context required an emphatic corrective focus accent the results suggest that stress clashes are not only resolved by stress or accent reduction, but that prominence is actually shifted onto the initial syllable even under focus marking and even when the clash is only present above the lexical level. The fact that stress shift was perceived without its shift triggering context also speaks against the claim that stress shift is a mainly perceptual phenomenon (cf. Grabe and Warren, 1995; Tomlinson et al., 2014). It has to be noted however, that the high intra-rater agreements but lower inter-rater agreement in determining stress shift indicates that listeners may have different strategies in determining word stress.

Furthermore, it was explored whether the possibility to shift the accented syllable away from the second focus accent leads to more double-accent productions. In Experiment 2, where such repair strategies were not available, speakers often omitted the first focus accent. The study found a correlation between stress shift and pitch accent placement. Most double-focus sentences had two prominences (ca. 75%), but the two conditions were affected differently. In the “clash” condition, there were more instances of two prominences when stress was shifted, and more instances of omitting the pitch accent on the first focused word when stress was not shifted. This suggests that alternation preferences may also interact with the prosodic marking of focus by pitch accent placement to prevent a pitch accent clash.

Concerning the *no clash* condition, where the adjacent pitch accents are separated by one syllable, the correlation of pitch accent placement and stress shift goes in the other direction - if stress is shifted, speakers more often omitted the first focus accent than when stress was not shifted, a so far unexplained phenomenon.

The fact that speakers produced two prominences in over 75% of the shiftable contexts of Experiment 3 but only in about 57% in the contexts of the experiment where stress shift was not an option (Experiment 2), however, does support the assumption that English speakers may have omitted pitch accents due to rhythmic reasons in the previous experiment and that the one unaccented syllable between the two focus accents often was not enough to dissolve the dispreferred clash.

## 5. General discussion and conclusion

The presented experiments investigated the interplay of alternation preferences and corrective focus marking in productions by German and English speakers. With German and English, two well-investigated stress-timed languages were chosen which share a number of similarities: They both have been found to prefer an alternation of strong and weak in production, perception and processing of sentences. They also both use pitch accenting to indicate focus structure, and they have been shown to do so by similar means. It has been found for both languages, however, that there is variation in the mapping of pitch accents and information-structure categories, and that we cannot assume a strict one-to-one relationship. Against this backdrop, this study investigated whether the preference for rhythmic alternation can account for variation in the prosodic marking of focus.

In both languages, the rhythmic manipulation in the experimental material does not seem to affect the placement of focus accents—there was no difference between the two double-focus conditions. However, the results with respect to pitch accent placement nevertheless differ between the two language groups. While the German speakers realized both focus accents by means of prominences in about 75% of cases (71% in clash and 79% in no clash sentences), the English speakers realized the two prominences in only about 56% of cases (in 51% of the clash and in 62 % of the no clash sentences.

It was assumed that this comparably lower number may be a consequence of the rhythmic environment after all, and no-clash sentences do not have enough material between the two foci in order to be rhythmically preferred. The higher degree of vowel reduction, and the frequent use of stress shift have been addressed as factors which might contribute to this difference between the two languages. The results obtained from the second experiment with English speakers on rhythm rule contexts indeed point in the direction of a rhythmic effect, since more focus accents were realized in both double-focus conditions when stress could be shifted.

However, the main difference in strategy between the two language groups manifests itself in the choice of pitch accent type in clash environments. German speakers often revert to a clash reduction in terms of melodic alternation—by realizing alternating pitch accent targets. Consequently, in a sequence of two focus accents they may realize the first one with a rising accent, that is with a low tonal target on the accented syllable (L*). This was not found for English speakers who almost always realized both foci with a high starred accent. English and German have been found to differ with respect to frequency of pitch accent types before. Mennen et al. (2012), for example, found a significantly higher number of L* accents in German than in English, while English speakers used H* accents more often than German speakers. This difference in frequency may be, possibly among other things, a consequence of the current finding that German speakers prefer an alternation of low and high, particularly in clash environments, and in general when the pitch accents are close together, as shown by Schauffler et al. (2015a).

The finding that pitch accent type can be adapted according to alternation preferences in German is another example of the “many to many mapping between prosodic form and discourse function” (Roettger et al., 2019), in other words, specific communicative functions that prosody has, such as signaling information structure, may be realized in several ways. This means that one discourse function may be realized with various accent types, or one accent type used for various discourse functions. The present study contributes to the identification of factors that bring speakers to choose one form over the other. Perception studies are needed in order to understand how listeners perceive and interpret these variants in different contexts.

Finally, it remains to be noted that we do find rhythmic readjustment also in focus contexts, that is, when the adjustable unit is focus marked, contrary to what has been previously claimed (Grabe and Warren, 1995; Hayes, 1995; Kentner, 2012b). It seems as if rhythmic readjustment or repair strategies on focus accents are not blocked because this focus carries the main sentence accent but because in single-focus sentences this is the last prominence in the phrase. The presence of a second sentence accent such as in double-focus sentences, however, generates adjustments with respect to pitch accent type in German, and with respect to pitch accent placement and stress shift in English.

With respect to the overall research questions, we can conclude that firstly, the preference for alternation can influence the prosodic marking of focus and is a source for variation in the realization of information-structure categories. The rhythmic context should therefore be taken into account when assigning semantic to phonological categories. And secondly, that even though German and English share the preference for alternation, it affects prosodic focus marking differently.

Future work will investigate how rhythmic repair strategies under focus marking are perceived, and what role speaker-specific strategies play.

## Data availability statement

The raw data supporting the conclusions of this article will be made available by the authors, without undue reservation.

## Ethics statement

Ethical review and approval was not required for the study on human participants in accordance with the local legislation and institutional requirements. The patients/participants provided their written informed consent to participate in this study.

## Author contributions

NS conceived, conducted, and analyzed the experiments and wrote the manuscript.

## Footnotes

^1^Wang and Féry (2018) refer to double-focus as dual-focus.

^2^In a third group of languages the crucial interval is ascribed to morae, which is why this group is referred to as “mora-timed” languages, such as, for example, Japanese.

^3^The effect was only found when the constituents did not vary in terms of animacy.

^4^Another repair strategy discussed by metrical accounts is lengthening of the stressed syllable so that a beat is added, and pause insertion (Liberman and Prince, 1977; Selkirk, 1984; Nespor and Vogel, 1986).

^5^Maintaining maximal contrast between adjacent units or units within short distances is not only beneficial for perception but also facilitates language production, as has been shown for example by Cutler in an experiment on speech errors (1980, as cited in Schlüter, 2005, p. 33).

^6^Jun (2012, 2014) terms these aspects of the tonal melody of languages “macro-rhythm” to differentiate it from the traditional speech rhythm based on weak and strong syllables, which she refers to as micro-rhythm.

^7^To control for frequency is important because prominence perception is not only signal-driven, but also expectation-driven, which means that the less predictable a word the more prominent it is, as found by Cole and colleagues (Cole et al. 2010, cited in Baumann and Winter 2018).

^8^See Schauffler (2021) for additional analyses on prominence relation and prosodic phrasing.

^9^A second analysis was conducted which keeps all differences in by creating a linear variable dividing the number of “prominence votes” by the number of all votes per item (nine). This resulted in a number between zero and nine representing the probability of a prominence rating. Taking this variable instead of the yes-no-variable that resulted from the four-out-of-nine-threshold for the statistical analysis did not change the result in any meaningful way. Therefore, the yes-no-variable was chosen since it is more representative of the task for the annotators who had to make a binary decision and could not judge prominence on a scale.

^10^This is comparable to other studies who found generally good agreement on whether or not a word carried a pitch accent. In Grice et al. (1996), for example, this agreement was reached in 87%.

^11^This case is from condition *clash* so that 54 recordings in the *clash* condition and 59 recordings in the *no clash* condition were analyzed.

^12^It remains to be seen how speakers realize the first focus accent if the second focus is not realized with an H*L but with a different accent type, as may be the case if itself is followed by a third focus.

^13^According to Kügler and Gollrad (2015), the meaning of contrast is conveyed by the height of the peak. They manipulated peak height and the leading rise to the peak (L+H* in GToBI), but did not consider the fall—in single-focus sentences the contrastive focus accent is typically the last pitch accent in the phrase and falls by the nature of being the last accent in the phrase (Féry, 2007).

^14^The mean number of syllables of the second NP in the context question was 3.8 and the mean number of syllables of the second object in the **clash** sentences was 3.9 (*t* = −0.2538, df = 37.77, *p* = 0.801) and in the no-clash sentences also 3.9 (*t* = −0.2582, df = 37.921, *p* = 0.7976).

^15^These categories may differ in fine phonetic detail across the two language groups (Atterer and Ladd, 2004 for example, found that pre-nuclear rises are aligned later in German than in English.). The phonetic implementation, however, is not under investigation in this study, so that the categorical labels are sufficient for our purposes.

^16^In an experiment on Mandarin Chinese, Kabagema-Bilan et al. (2011) found something similar, namely that the first focus in a double-focus context was not marked with increased F_0_ and duration when there was only one syllable between the first and the second focus, which points toward an effect of the rhythmic context.

^17^The prominence annotation for the verbs is, however, solely based on the author's impression, so that further testing is needed in order to understand whether the shift of prominence to other material outside the focus domain preceding the focus is a systematic strategy in clash or “near-clash” environments.

^18^See Schauffler et al. (2019) where the results of this experiment were compared to realization of the material by English L2 speakers.

^19^The lower inter-annotator agreement was mainly caused by one of the German annotators who often differed in her perception. A second analysis excluding this annotator did not yield different results in any meaningful way.
